# Supplementary material for: Identification of natural Zika virus peptides presented on the surface of paediatric brain tumour cells by HLA class I
Source: PLoS One. 2025 Oct 30;20(10):e0335726. doi: 10.1371/journal.pone.0335726 (PMC12574884; doi:10.1371/journal.pone.0335726)
Supplement: Attachment — Submitted filename: PLOS One Response-to-reviewers-final.pdf [file pone.0335726.s003.pdf]

We thank the reviewers for their detailed comments and provide a point-by-point response below.

Reviewer #1: In this paper the authors employ a proteomic analysis to identify fragments of Zika peptides presented by MHC on brain cancer cells infected with Zika. The paper seems mostly descriptive and narrow in scope, but may be an important resource for the field (see points below).

Reviewer #2: In the manuscript#PPATHOGENS-D-24-01982, entitled "Identification of natural Zika virus peptides presented on the surface of paediatric brain tumour cells by HLA class I", the authors performed proteomic profiling for HLA-presentation of ZIKV-derived peptides using a human tumor cell line—USP7-ATRT which is an aggressive pediatric atypical teratoid rhabdoid tumor cell line previously shown to be susceptible to Zika viral infection and oncolysis. They identified 19 Zika virus-derived peptides, with 17 of them predicted to have strong binding affinities to USP7 cell's HLA-I molecules, and among them 13 peptides were predicted to be immunogenic. The most abundant and immunogenic peptides were predicted to be derived from NS3-viral protein. While the study has a limited scope, it is significant and provides key information about ZIKV-immunogenic peptides in a human brain tumor cell line. Earlier work by this group and others have indicated that ZIKV infection induces oncolysis of pediatric brain tumor cells in vitro and in vivo, and triggers immune responses against spontaneous brain tumors in canines. Zika virus's neurotropism while causing self-limiting acute infection in children offers a new approach for targeting pediatric CNS tumors via oncolytic virotherapy.

Reviewer #3: The presented manuscript by Sherwood et al. reports the identification of HLA presented peptides from a Zika virus infected brain cancer cell line.

While the manuscript provides important insights into the presented peptidome in the context of a single brain tumour cell line that expresses three HLA class I alleles, some of its conclusions are based on preliminary data, and premature at times. The manuscript does not experimentally explore functionality and immunogenicity of the presented peptides other than by a run through an existing immunogenicity predictor, and a blast search against the human predicted peptidome.

Major comments

Reviewer #1:

The authors identify fragments of Zika peptides presented by HLA on brain cancer cells. They identify 19 Zika peptides, helping them to understand the specificity of the human T cell response against Zika, which could help mediate the antitumoral efficacy of Zika as OV therapy.

The authors mention that the significance of their study is that knowing the specific Zika peptides that are presented may help to develop novel brain tumour immunotherapies, although I had a hard time understanding exactly how this information would lead to enhanced immunotherapies. First, different people may have different MHC alleles, so some of these peptides may just not be recognized in all people. Second, the the OV activity of Zika and other OV viruses is not only dictated by the specificity of the T cell response, but also innate pathways that trigger inflammation/costimulation/influx of immune cells to the Tumor Microenvironment. This should probably be discussed more.

- To address the first point, we have added a column to Table 1 detailing the percentage of the human population that expresses the HLA allotypes that we identify in USP7-ATRT. Additionally, we have added the following text to the Results: "USP7-ATRT cells express the globally most common HLA-A allotype (HLA-A*02:01), which is expressed by nearly 40% of the human population (Table 1). Additionally, USP7-ATRT expresses HLA-I molecules HLA-B*44:02 and HLA-C*05:01, and the HLA-II molecule HLA-DRB1*15:01, which are expressed by 7.61%, 7.85% and 18.41% of the human population, respectively (Table 1)."
- To address the second point, we have added the following text to the Introduction: "The second pillar of oncolytic virotherapy is the mounting of anti-tumoral immune responses following immunogenic cell death (ICD) of cancer cells. Tumour antigens, viral antigens, cytokines, pathogen-associated molecular patterns (PAMPs), and damage-associated molecular patterns (DAMPs) are released into the tumour microenvironment (TME) during OV-induced ICD, and this leads to inflammation (5). Initially, these factors recruit and stimulate innate immune cells such as monocytes, macrophages, dendritic cells (DCs), neutrophils, and natural killer (NK) cells. Professional antigen-presenting cells (APCs) such as macrophages and DCs bridge innate and adaptive immunity by processing and presenting tumour and viral antigens on their cell surface. Adaptive immune system cells such as T cells migrate along chemokine and molecular gradients to locate, infiltrate and drive an adaptive immune response against the infected tumour. Thus, the efficacy of OV therapy arises from various components, including oncolysis, the innate immune response and the adaptive immune response."

They analyze peptides that bind to USP7-ATRT brain tumour cells, which express HLA-A allotype (A*02:01) that is widely expressed in the population, but there is still variation in there MHC alleles expressed in humans, so I am not sure how widely generalizable the epitope mapping studies are when using a cell line.

- We believe that the column added to Table 1 (detailed above) addresses the issue raised here.

However, the paper may be an important resource and thus worthy of publication, if they address the above limitations.

Also, could there authors please discuss more that the use of Zika as an OV can raise safety concerns?

- We have added the following paragraph to the Discussion to address this: "Safety concerns must be addressed for ZIKV OV therapy to progress to clinical trials, including (i) its potential for neurotoxic off-target effects and (ii) the concern of administering a replication-competent OV to immunosuppressed cancer patients. Several studies report preferential targeting of ZIKV for tumour cells over normal neural cells, and this is exemplified in canines bearing natural brain tumours where tumour tropism, but not neurotoxicity, was observed (19,20,36,37). Recently, members of our research team genetically engineered ZIKV to express a miRNA response element that sensitises the virus to non-tumour miRNA, thereby negating ZIKV neurotoxicity by reducing viral replication in normal cells (38). The immunodeficient nature of xenograft mice partly models the immunosuppressed nature of cancer patients. Intratumoural ZIKV administration to xenograft mice bearing brain tumours is asymptomatic with limited and non-permissive viral shedding, and does not cause adverse effects or neurological

damage (19,20). However, viral shedding and the infection of normal cells and tissues in a murine model do not fully recapitulate that of the human body. Consequently, genetic engineering of ZIKV is necessary to improve its safety profile, and current approaches have predominantly focused on employing small nucleotide changes or repurposing live attenuated vaccines (18,23,38–41)."

Reviewer #2:

1- While MS analysis provides comprehensive proteomic profiling, flow/spectral cytometry would be important to validate the key proteomic findings, such as the upregulated expression of HLA-I antigen processing and presentation-related genes after ZIKV infection of brain tumor cells. This would help confirm the results beyond the USP7 cell line by analyzing primary brain tumor cell samples.

- We thank the reviewer for their suggestion and whilst we agree that cytometry would help to validate if ZIKV upregulates the HLA-I pathway, our observations at the global proteome level were sufficient for us to hypothesize that USP7-ATRT cells would present ZIKV peptides. Thus, we decided to focus on investigating the specific ZIKV peptides presented by this pathway, rather than further characterization of the ZIKV effect on the pathway. Although beyond the scope of the current study, we recognize, as the reviewer suggests, that analysis of ZIKV-infected primary brain tumor samples would add to our cell-based in vitro work here.

2- Similarly, in vitro HLA binding assays of the identified immunopeptides are essential to validate HLA binding affinity predictions from the NetMHC tool.

- We agree that in vitro HLA binding would add further to the research we perform here. First, some of the ZIKV peptides that we have identified have been validated in terms of their immunogenicity, and we provide references to these in the paper. Whilst it would be ideal to validate all peptides in vitro, we contend that predicted binding affinities are becoming increasingly widespread along with the improvement of machine-learning methods and generation of datasets for testing. Affinity predictions are frequently reported in the literature, we believe that it is acceptable to publish the stand-alone NetMHC predictions at this point.

3- Upregulation of antigen processing and presentation is among the common downstream effects of viral infection-induced IFN I responses. Do the authors observe a general activation of IFN I-stimulated genes after ZIKV infection in USP7 cells?

- Based on our gene ontology, KEGG and Reactome enrichment analyses of ZIKV-infected USP7-ATRT cell RNA-Seq, we do not observe upregulation of Type I IFN responses at the biological process/pathway level. However, at the single gene level, we do observe significant upregulation of genes such as IFIT1 and IFIT2. This data is available to view in Figures 2 and 3 of our recent preprint titled "Multi-omics analysis reveals key immunogenic signatures induced by oncolytic Zika virus infection of paediatric brain tumour cells" (https://www.biorxiv.org/content/10.1101/2024.11.28.625843v1)

4- Lack of HLA-II-presented peptides due to the use of the USP7 cell line limits the scope of understanding how ZIKV infection triggers both CD4 and CD8 T cell responses in the brain. Adding dendritic cells to the infection culture followed by proteomic analysis may help address this gap.

- We agree with the reviewer – and appreciate the suggestion. Although we have focused on HLA-I in this report, future work could extend to HLA-II using similar approaches. We have acknowledged this as a limitation to the current study in the Discussion. We believe that publishing our work with a specific focus on HLA-I, and thus CD8+ T cells, holds merit as CD8+ T cells are principal contributors to ZIKV-induced tumour clearance.

5- Minor comment: The authors used RNA-seq data of the USP7 cell line for HLA typing. While it is a feasible approach for preliminary identification of HLA allotypes, orthogonal methods such as PCR-based HLA typing or targeted sequencing may be needed to validate the results.

- The reverse HLA typing (unsupervised clustering, MixMHCp) that we perform from the sequences of approximately 3,000 9mers (Figure 2B) is in perfect agreement with the RNA-Seq HLA typing. We believe that this adds weight to the RNA-Seq HLA typing. We also recognize that RNA-Seq HLA typing workflows are becoming widely used.

Reviewer #3:

The writing of the manuscript seems rushed, abbreviations are not properly introduced, and there are a few essential and specific suggestions from my side:

(i) HLA-I locus was typed from RNAseq - why not class II if the aim was to analyse both? What was the rationale for analysing class II? Is there any evidence that the USP7ATRT cells are class II positive in the RNAseq data? Where did the authors retrieve the result that the cell line is DRB1*15:01 positive? A pan-class II antibody was used for the experiments, so typing of the other gene loci would have been important. No analysis on the class II data is presented, and therefore no claim can be made that they are actually class II-derived peptides. What was the percentage of predicted binding to DRB*15:01?

- Both HLA class I and II were typed from the RNA-Seq data, and this is where we identified that USP7-ATRT are positive for DRB1*15:01 (Table 1). Following our observation that USP7-ATRT expressed both HLA class I and II, we set out to assess the capability of USP7-ATRT cells to present ZIKV peptides by both HLA class I and II. However, following the low recovery of HLA class II peptides with the pan-class II antibody, and since we did not observe any ZIKV peptides which were pulled down by the pan-class II antibody, we decided to focus our analysis on HLA class I. The HLA-II peptides were those that we recovered by immunopeptidome pulldown with the pan-class II antibody, and that is why we claim that they are class II-derived peptides (Table 2 and Figure 2A).

(ii) I doubt that USP7ATRT cells are homozygous for HLA-A*02:01, but rather that the cell has lost heterozygosity, as common for tumour cell lines. This might extend to the class II locus.

- We thank the reviewer for their insight and have addressed this by adding the following to the Results: “This analysis indicates that USP7-ATRT cells are homozygous for all three classical HLA-I alleles, but this may be a byproduct of HLA heterozygosity loss during transformation.”

(iii) It is interesting that the authors detect a relationship between viral protein abundance and immune presentation, however, a more formal analysis is needed. It is not clear whether there is an actual correlation between the two from the figures alone, and a detailed correlative score

and analysis is missing. It would have been helpful if the authors would perform several analyses here, to establish whether HLA peptide counts correlate with the proteomics rankings (which, by the way, are not an absolute measure of protein quantity at all), or whether it is HLA-binding rank score that correlates better. Limitations of the chosen approach, particularly in view of the protein ranking estimate, must be discussed carefully. It should be described in the main text more accurately how the correlation analyses have been performed. Since protein abundance and correlation with HLA peptide presentation is broadly absent in other systems, more evidence is needed here to claim the scientific result. For example, it would be important to understand how the HLA peptide presentation relates to the transcript abundance, which has been shown to often more directly correlate with HLA presentation if compared to quantities of protein. Table 3 is obsolete, in my view.

- We thank the reviewer for their suggestion and have readdressed our claims of there being a "correlation" and have now referred to this relationship as a "trend". The relationship between ZIKV protein abundance and peptide presentation is an interesting sidenote to the main work we perform here, and as such, we only wanted to highlight it briefly to the reader. In the absence of performing correlative analysis, we have left Table 3 in the manuscript to assist with the reader assessing the trend between viral protein abundance and immunopeptide number

(iv) Finally, the authors claim that the Zika virus peptides do not mimic self-peptides by comparing their sequences against the human proteome. Presumably, this exercise is supposed to support the hypothesis that the viral peptides would be immunogenic? The conclusion that viral peptides do not mimic human self-peptides is premature, and cannot be made on the basis of the presented analysis - not only because many sequences that may be presented by HLA in vivo may not be considered by the author's approach, including those from canonical regions.

- Yes, we performed this analysis to support the hypothesis that viral peptides would be immunogenic, but crucially also to see if the ZIKV peptides may have autoimmune implications by mimicking human peptides. We have readdressed our statements regarding this analysis and have carefully included that these are simply predictions. We acknowledge the limitation of this approach raised by the reviewer and have added the following text to the Discussion: "A limitation of this prediction is that the alignment is against theoretical peptides of the human proteome and, therefore, may not encompass all human peptides that may arise in vivo."

Minor Issues: Editorial and Data Presentation Modifications

Reviewer #1: I suggest to use complete words. The character "&" could be replaced for the word "and" unless there is a specific reason for using the & character (e.g. antigen processing & presentation, used throughout the text).

- The "&" character has now been replaced with "and" for all instances other than when the exact name of the "Class I MHC mediated antigen processing & presentation" Reactome pathway is mentioned, as this pathway name specifically uses the "&" character.

Reviewer #2: (No Response)

Reviewer #3: The sentence in the authors summary "Zika virus (ZIKV) uses both mechanisms to 60 fight brain cancer." does not make sense - the virus is not "fighting" the cancer actively, it rather renders the cells immune-recognisable. Please reformulate.

- We have readdressed the sentence to say: “ZIKV uses both mechanisms to induce cancer cell death and render the tumour recognisable by the immune system.”

If you use the abbreviation USP7 for USP7-ATRT cells, please introduce it carefully, as the abbreviation is identical to the name of the USP7 protein. I would suggest not to abbreviate the cell line name to avoid confusion.

- All text references of USP7 have now been changed to USP7-ATRT, and abbreviations have been added to the end of figure legends.

Method section: Please correct notation of "degrees celsius", which is currently written either "oC" or "C".

- All instances have been corrected to °C.

I did not understand Figure 1, and clarification is needed. The subfigures are not in order (D before C) and legend does not explain figure 1C appropriately, nor does it explain all columns shown in tables B/D. In fact, tables should be added as tables in the manuscript, and information on proteins defined in the identified pathways should be listed, together with their regulation factors.

- The Figure legend has been improved, and Figures 1B and 1D have been refined for clarity. Figures 1B and 1D remain in the same order to aid the visualisation of Figure 1 and the comparison between the two results. All genes which contributed to the leading edge of the GSEA enrichment analysis have been listed in Supplementary Table 1.
