# Supplementary material for: Identification of natural Zika virus peptides presented on the surface of paediatric brain tumour cells by HLA class I
Source: PLoS One. 2025 Oct 30;20(10):e0335726. doi: 10.1371/journal.pone.0335726 (PMC12574884; doi:10.1371/journal.pone.0335726)
Supplement: Attachment — Submitted filename: response-to-reviewers.docx [file pone.0335726.s004.docx]

Response To Reviewers Round 2
Reviewer #1

***My specific concerns are:***

1. *Regarding the identification of MHC pathway enrichment by GSEA in proteome and public transcriptome analysis, authors restricted their reference pathway database to an immunity Reactome module, potentially increasing the chance of spurious enrichment. In that case a stricter P and FDR threshold would be adequate.*

We recognize the reviewer’s concerns and note that with different content, and statistical methods, different enrichment analysis approaches can give differing results. In our case, using all of Reactome (rather than just the immunity module) did not provide statistically significant pathway terms after multiple-testing. However, we have also performed new analysis to show that these immune-related categories are significant and not spurious. Taking the gene set identified as significantly (p<0.05) elevated in the global proteome analysis at 24hrs after ZIKV infection, we see that enrichment analysis of several other pathway databases score ‘Immune System’, ‘T-cell activation’ and ‘Antigen Presentation’ as ranking very highly (2^nd^ most enriched category for example) and with statistical significance. This has been detailed in the first paragraph of the Results section and now leads into the analysis previously presented using the Reactome Immunity module.

2. *Somewhat related to MHC enrichment, flavivirus including ZV are known to inhibit type I IFN´s production and effect. Type I IFN are known potentiators of MHC expression and antigen presentation. These interactions in the context of brain tumors and the current model should be discussed.*

This is an important point – and we agree that the interplay between Zika virus (ZIKV), interferon signalling and MHC activity and antigen presentation is complex and also relevant to our findings and to future oncolytic virotherapy development. As the reviewer notes, ZIKV, along with other flaviviruses can antagonize type I IFN signalling and thereby affect MHC Class I activity and the repertoire of presented peptides. We’ve added to paragraphs 3 and 4 of the Discussion to discuss these points.

3. *19 ZV peptides out of 3,658 (Table 2) correspond to 0.5 % of the MHC-I-eluted peptides. Is this what is expected assuming MHC-I pathway enrichment? Without a ZV-susceptible non-tumoral cell as reference, the main finding is difficult to interpret.*

Yes, this is within the range found in other studies where typically ~1% of the MHC peptidome will be derived from the virus. Although there are no comparable Zika virus studies, vaccinia virus studies indicated that the MHC-1 peptidome was <1% viral derived (<https://pmc.ncbi.nlm.nih.gov/articles/PMC6386720/>), and ~0.6% for human coronavirus (<https://pubmed.ncbi.nlm.nih.gov/37498934/>). Also, similar numbers to a study with papillomavirus (<https://doi.org/10.1002/pmic.201700390>) and a previous study of the influenza A immunopeptidome from members of our group (<https://journals.plos.org/plospathogens/article?id=10.1371/journal.ppat.1009894>).

4. T*he cell line USP7-ATRT as model: Although a CNS neoplasia, atypical teratoid/rhabdoid tumors are highly undifferentiated tumors with high histologic diversity combining rhabdoid, neuroectodermal, epithelial, and mesenchymal elements. Authors describe their model as a proxy of neuronal precursor, the normal target of ZV, however it’s not clear which are the neuronal features of the USP7-ATRT cell used as model.*

We appreciate the reviewer’s interest in the USP7-ATRT cell line. This cell line was established from a left temporal supratentorial ATRT tumor resected from a three-year-old patient. Molecular analyses have demonstrated that USP7-ATRT retains a SMARCB1/INI1-positive profile, consistent with a subtype of ATRT closely related to normal neural stem cells, as previously reported (Ho et al., BMC Med Genomics, 2015). Notably, USP7-ATRT cells robustly express canonical neural stem cell markers, including Nestin, SOX2, and Musashi-1. Furthermore, global gene expression profiling revealed a strong transcriptomic resemblance to normal neuroprogenitor cells. This includes a conserved expression pattern of TAM receptor genes and other host factors implicated in ZIKV entry. A comprehensive phenotypic and molecular characterization of the USP7-ATRT cell line has been published in detail (Kaid et al., Cancer Res, 2018).

References:

Ho DM, Shih CC, Liang ML, Tsai CY, Hsieh TH, Tsai CH, Lin SC, Chang TY, Chao ME, Wang HW, Wong TT. Integrated genomics has identified a new AT/RT-like yet INI1-positive brain tumor subtype among primary pediatric embryonal tumors. BMC Med Genomics.

2015 Jun 25;8:32. doi: 10.1186/s12920-015-0103-3. PMID: 26109171; PMCID: PMC4480900.

Kaid C, Goulart E, Caires-Júnior LC, Araujo BHS, Soares-Schanoski A, Bueno HMS, Telles-Silva KA, Astray RM, Assoni AF, Júnior AFR, Ventini DC, Puglia ALP, Gomes RP, Zatz M, Okamoto OK. Zika Virus Selectively Kills Aggressive Human Embryonal CNS Tumor Cells In Vitro and In Vivo. Cancer Res. 2018 Jun 15;78(12):3363-3374. doi: 10.1158/0008-5472.CAN-17-3201. Epub 2018 Apr 26. PMID: 29700002.

5. *The importance of the HLA-A*02:01 allele: Authors put a considerable effort in providing evidence that the HLA-A*02:01 allele expressed in USP7-ATRT cells is highly frequent globally, as if no other alleles could also bind ZV-derived peptides and induce CTL response. In contrast with low complexity peptide vaccines of single protein vaccines, were making sure that the potential peptides can be presented by MHC in the highly diverse human population, the use of a live virus is a different scenario: There are at least several antigenic viral proteins, which are likely to be presented by different HLA genes and allele combinations in the global population.*

   *Thus, the argument of HLA-A*02:01 allele being the most frequent in the global population is not necessary, as it is also unnecessary to estimate the binding affinities of the identified peptides with the HLA-A*02:01 allele. In fact, it would be more informative to estimate binding affinities of the identified peptides with other globally common HLA alleles.*

   *Moreover, authors should provide the exact source of the HLA-A*02:01 allele frequency reported in table 1. Searching the Allele Frequency Net Database http://www.allelefrequencies.net/, HLA-A*02:01 allele is more frequent in European population (but not 40%, as stated), but not so in other major populations. Also, reported frequencies are subjected to strong population sampling biases, being the European population the most sampled.*

We agree with the reviewers concerns and thank them for raising these points. We have revised the manuscript to de-emphasise the importance of HLA-A*02:01 and updated the reference of the global variance in HLA-A*02:01 frequency. Whilst we believe that studying cell lines expressing common HLA allotypes is of clinical relevance, for the widest application, of greater importance are insights into which Zika proteins and which regions of the Zika proteins are most likely to be processed and presented by HLA in general. Specifically, we have updated the HLA allele frequencies reference to a more recent 2020 study (DOI:10.1111/tan.13811), and revised the manuscript at lines 49, 133, 199, 218 (Table 1), 327, 435.

6. **Minor concerns:**
1. L*ine 105. “via vertical transmission (12)”. I suggest changing to “transplacental transmission”, since vertical transmission implies the transmission of genetic information through the germline.*

The text has been adjusted to say “transplacental transmission”, as recommended by the reviewer.

2. *Line 293. “Downregulating HLA-I presentation is a method…”. I suggest changing to “mechanism”.*

The text has been adjusted to say “mechanism”, as recommended by the reviewer.

3. *Microglial cells have antigen-presenting functions via class II and are targets of ZV. Should be discussed in the context of oncolytic virus therapy.*

The following section has been added to the Discussion where we discuss HLA-II-presentation: “Microglia are the primary immune cells of the CNS, can be infected by ZIKV, and alongside DCs, possess antigen-presenting capabilities (33). In immunocompetent mice GL261 glioma models, ZIKV administration led to increased infiltration of MHC-II-expressing microglia in the primary tumour and in long-term survivors following glioma rechallenge (22,23). Thus, how microglia antigen presentation contributes to, and how microglia interact with CD4+ T cells during, the oncolytic response of ZIKV requires further investigation.”

## Reviewer #2

1. *First, no T cell assays (e.g., ELISpot or cytotoxicity assays) are included to confirm the immunogenicity of the identified peptides. Although the authors reference previous studies, would it be feasible to include such data to directly demonstrate T cell activation in response to the novel peptides identified here?*

We thank the reviewer for their positive comments. We agree that validating the immunogenicity of the viral peptides in the laboratory using the assays suggested would be a next step in confirming the immunogenicity of the peptides. This is currently out of the scope of the project, but we have acknowledged this limitation in the Discussion.

2. *Additionally, immunopeptidomics was performed only once (line 417), which limits the reproducibility of the peptide identification, and the manuscript does not acknowledge this limitation. The findings are also based on a single cell line (USP7-ATRT), which, while appropriately justified as a relevant model, restricts the generalizability of the results. Could the study be extended to include additional cell lines? Another limitation is the lack of experimental validation for affinity and stability of HLA-peptide binding, which is based solely on computational prediction.*

We have added text to highlight the limitation of only investigating one cell line in our study and acknowledge the limitation of performing the analysis on a single sample. As the reviewer notes, this limits the reproducibility and generalizability of our study. However, increasingly meta-analysis and machine-learning approaches are being used to combine individual immuno-peptidomics studies where sample sizes may be limited. Publishing and depositing (in databases such as PRIDE) studies such as ours can contribute to these meta-analyses. Also, although our study is more descriptive than quantitative, the data do reveal peptides identified in common with Dengue studies and also highlight common features of other flavivirus immunopeptidomes such as the enrichment of peptides derived from NS3.

We acknowledge that the lack of experimental validation for the HLA-peptide binding limits the impact of our research, predicted binding affinities are becoming increasingly widespread along with the improvement of machine-learning methods and the generation of datasets for testing. Affinity predictions are frequently reported in the literature, and we believe that it is acceptable to publish the stand-alone NetMHC and other predictions at this point.
